# Targeted Clinical Metabolite Profiling Platform for the Stratification of Diabetic Patients

**DOI:** 10.3390/metabo9090184

**Published:** 2019-09-14

**Authors:** Linda Ahonen, Sirkku Jäntti, Tommi Suvitaival, Simone Theilade, Claudia Risz, Risto Kostiainen, Peter Rossing, Matej Orešič, Tuulia Hyötyläinen

**Affiliations:** 1 Steno Diabetes Center Copenhagen, 2820 Gentofte, Denmark; la@biosyntia.com (L.A.); tommi.raimo.leo.suvitaival@regionh.dk (T.S.); stheilade@hotmail.com (S.T.); claudia.risz@chello.at (C.R.); peter.rossing@regionh.dk (P.R.); 2 Drug Research Program, Division of Pharmaceutical Chemistry and Technology, Faculty of Pharmacy, University of Helsinki, 00014 Helsinki, Finland; sirkku.e.jantti@gmail.com (S.J.); risto.kostiainen@helsinki.fi (R.K.); 3 Department of Clinical Medicine, University of Copenhagen, 1165 Copenhagen, Denmark; 4 Turku Centre for Biotechnology, University of Turku and Åbo Akademi University, 20520 Turku, Finland; matej.oresic@oru.se; 5 School of Medical Sciences, Örebro University, 702 81 Örebro, Sweden; 6 Department of Chemistry, Örebro University, 702 81 Örebro, Sweden

**Keywords:** clinical diagnostics, diabetes, metabolomics, mass spectrometry

## Abstract

Several small molecule biomarkers have been reported in the literature for prediction and diagnosis of (pre)diabetes, its co-morbidities, and complications. Here, we report the development and validation of a novel, quantitative method for the determination of a selected panel of 34 metabolite biomarkers from human plasma. We selected a panel of metabolites indicative of various clinically-relevant pathogenic stages of diabetes. We combined these candidate biomarkers into a single ultra-high-performance liquid chromatography-tandem mass spectrometry (UHPLC-MS/MS) method and optimized it, prioritizing simplicity of sample preparation and time needed for analysis, enabling high-throughput analysis in clinical laboratory settings. We validated the method in terms of limits of detection (LOD) and quantitation (LOQ), linearity (*R*^2^), and intra- and inter-day repeatability of each metabolite. The method’s performance was demonstrated in the analysis of selected samples from a diabetes cohort study. Metabolite levels were associated with clinical measurements and kidney complications in type 1 diabetes (T1D) patients. Specifically, both amino acids and amino acid-related analytes, as well as specific bile acids, were associated with macro-albuminuria. Additionally, specific bile acids were associated with glycemic control, anti-hypertensive medication, statin medication, and clinical lipid measurements. The developed analytical method is suitable for robust determination of selected plasma metabolites in the diabetes clinic.

## 1. Introduction

The incidence of type 2 diabetes (T2D) is rising globally, currently estimated to exceed 450 million patients worldwide. In addition, the prevalence of prediabetes is approximately two to three times higher than for diabetes. Prediabetes is a condition with a high risk of progression to T2D, with a yearly conversion rate of 5–10% [1,2]. It is also known that excessive hepatic fat accumulation is a typical feature of T2D patients and plays an important, pathogenic role in disease development and progression. Particularly, non-alcoholic fatty liver disease (NAFLD) may have an important, deleterious impact on diabetic patients, increasing the risk of cardiovascular complications. Moreover, there is evidence of associations between prediabetes and complications of diabetes such as early nephropathy, small fiber neuropathy, early retinopathy, and risk of macrovascular disease [2]. Therefore, there is a need for predictive tools for efficient and accurate tracking of the progression from the state of normal glucose tolerance (NGT) to prediabetes and finally to T2D, as well as a need for the identification of those individuals with T1D and T2D who are at risk of developing diabetic complications. There is also a need for improved stratification of those individuals who already have the disease based on their risk of developing complications. Finally, there is a pressing need to then tailor intervention strategies to these individuals. Ideally, knowledge about the underlying pathophysiological characteristics associated with either fasting or postprandial glucose dysregulation would be utilized in order to optimize the efficacy of any interventions [3].

The complex etiology of diabetes makes effective screening, diagnosis, prognosis, and intervention challenging [4]. Several studies have shown changes in the circulating levels of specific metabolites prior to an individual developing overt T2D. For example, the Framingham Offspring, European Investigation into Cancer and Nutrition (EPIC) Potsdam, Metabolic Syndrome in Men (METSIM), Cardiovascular Risk in Young Finns (CRY), and Southall and Brent Revisited (SABRE) studies have replicated the finding of increased levels of branched-chain amino acids and their derivatives, aromatic amino acids, even years ahead of conversion to overt T2D [5,6,7,8,9,10]. Amino acids, particularly tyrosine, were found to be associated with risk of microvascular disease [11]. Additionally, other metabolites (e.g., 1,5-anhydroglucitol, norvaline and l-aspartic acid) were found to be associated with macroalbuminuric diabetic kidney disease [12,13], while glutamine, glutamic acid, and symmetric dimethylarginine (ADMA) were suggested as potentially-predictive biomarkers of diabetic complications [14,15,16]. Several metabolites (e.g., β-hydroxypyruvate and 1,5-anhydroglucitol (1,5-AG)), were associated with regulation of glycemic control [17,18]. Many lipids were identified as predictive biomarkers of diabetes. Specifically, triglycerides of low carbon number and double-bond count as well as lysophosphatidylcholine, LPC(18:2), were identified as early predictors of T2D [19,20]. Notably, these markers were unaffected by obesity [19]. Additionally, bile acids has been associated with T2D and insulin resistance [21,22]. Mannose [23], 2-aminoadipic acid [24,25], as well as indoxyl-sulfate and cresyl-sulfate [26] were suggested as possible biomarkers and creatinine [27] is already routinely implemented as an estimate of renal function. In addition to creatinine, several other metabolites, mainly amino acids and lipids, have been suggested as specific biomarkers for early diagnosis and assessment of the diabetic kidney disease (DKD), as summarized in a recent meta-analysis [28,29].

Most of the studies described above have been performed with non-targeted metabolomics methods, using workflows which are difficult to apply in routine clinical laboratory settings. Herein, our goal was to develop a fast and robust method for quantitative analysis of a selected panel of metabolite biomarkers, which are informative as to the prediction and diagnosis of (pre)diabetes and its co-morbidities/complications, as well as in follow-up of interventions. We developed a method which includes 34 metabolites, representing several metabolite classes, including amino acids, bile acids, carnitines, phenolic compounds, and small organic acids. The method is based on simple sample preparation and fast, quantitative ultra-high-performance liquid chromatography coupled to tandem mass spectrometry (UHPLC-MS/MS) analysis. Both sample preparation and the subsequent analyses were optimized and validated. Additionally, the method was demonstrated in a subset of samples from a cohort of diabetic patients, who were observed at the Steno Diabetes Center Copenhagen between 2009 and 2011 [30].

## 2. Results

Based on our earlier diabetes-related studies, as well as on the results published in the literature, we selected 34 specific metabolites for this study (Table 1, Supplementary Materials, Figures S1–S3) [2,5,6,7,8,10,11,14,17,18,19,20,21,23,24,28,31,32,33,34]. Our aim was to develop a robust and fast analytical assay in terms of both sample preparation and analysis, for quantitative determination of these selected metabolites. However, analyzing both highly polar and nonpolar metabolites in a single method is highly problematic. As some of the candidate biomarkers (e.g., very polar sugar derivatives and neutral lipids such as triacylglycerols) would have required a second sample preparation step and/or analytical method, these were excluded from the final method. The method was validated in terms of (a) limit of detection (LOD), (b) limit of quantitation (LOQ), (c) linearity (*R*^2^) and linear range, and (d) intra- and inter-day repeatability of each analyte.

### 2.1. Sample Preparation

Here, we combined a simple protein precipitation with acid followed by derivatization of amino acids and structurally-related compounds (Figure S4). For the protein precipitation, acidic conditions were chosen, as protein precipitation with methanol or acetonitrile would have required evaporation of the solvent prior to derivatization and analysis. The amount of derivatization reagent, the amount and type of the solvent and buffer as well as the time for the derivatization reaction were optimized. Since the derivatization reagent has an impact on the MS detection, the conditions were optimized to decrease ion suppression as well as to improve the overall robustness of the method. Dry ACN was used for dissolving the AQC-reagent, as even trace amounts of water in the solvent can react with the reagent. The final sample preparation conditions included protein precipitation with SSA, followed by neutralization and pH adjustment using a mixture of carbonate buffer and NaOH) prior to the derivatization with AQC in anhydrous ACN. The MS spectra showed that only amino acids and related compounds with amino acid functionality (namely the amino acids, AADA, ADMA, SDMA, kynurenine, and taurine) were derivatized and not any of the other targeted compounds.

### 2.2. LC-MS

MS- and MS/MS-spectra were acquired for each of the analytes in order to select optimal precursor and product ions for selected reaction monitoring (SRM) analyses (Figures S5–S9). Depending on the ionization properties of the different analytes, protonated ([M+H]^+^) or deprotonated ([M–H]^−^) molecules were chosen as precursor ions. MS/MS-spectra were acquired and the most selective and intense product ions were selected for SRM analyses. When possible, one ion transition was chosen for quantification and another ion transition was chosen as the qualifying ion transition to ensure correct measurements of the analytes. Finally, the analysis parameters (fragmentor voltage, collision energy, cell accelerator voltage) were optimized for each ion transition (Table 2). All the derivatized amino acids and related compounds produced the product ion [M-H-170]^−^. These were then selected for SRM analyses together with one other diagnostic product ion (where possible). Among the bile acids, CDCA and UDCA were not fragmented and; therefore, the only chosen product ions for these two analytes were their deprotonated molecules. For isomeric compounds (GCDCA, GDCA, and GUDCA; TCDCA, TDCA, and TUDCA) the MS and MS/MS-spectra are similar to the same three main product ions and their separation depends on chromatographic separation. In addition, TCA shows the same three main product ions as TCDCA, TDCA, and TUDCA, but has different precursor ions.

**Figure 1:**
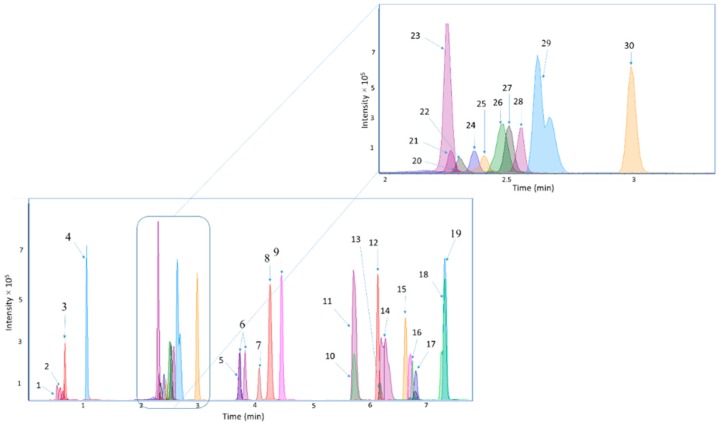
Chromatograms representing the chromatographic separation of the analytes. The peak numbers correspond to the following analytes: (1) Crea, (2) GBB, (3) β-OHB, (4) N-MNA, (4) Kynu, (6) Leu and Ile, (7) Phe, (8) AzelA, (9) Trp, (10) TUDCA, (11) TCA, (12) GCA, (13) GUDCA, (14) TDCA and TCDCA, (15) CA, (16) CDCA, (17) GCDCA and GDCA, (18) UDCA, (19) DCA, (20) Gly, (21) Gln, (22) ADMA and SDMA, (23) taurine, (24) Phe, (25) Gln, (26) HCit, (27) Ala, (28) AADA, (29) IndS and (30) Tyr.

In the optimization of the LC-MS method, different columns (Ascentis Express RP-Amide, Poroshell 120 SB-AQ, Acclaim RSLC PolarAdvantage, Acclaim Trinity P2, and Kinetex^®^ F5 column) and different LC modes were tested. Based on the resolution of the chromatographic separation, the Kinetex^®^ F5 column was chosen for further optimization. The conditions were optimized to include sufficient retention for the most polar compounds, and a short overall analysis time. Therefore, the gradient elution was initiated at 99% of the aqueous eluent. The UHPLC method showed good chromatographic performance (Figure 1), fulfilling general acceptance criteria for an analytical method (Section 4.4). For a few of the analytes, the resolution was; however, insufficient to achieve baseline separation and due to very similar MS/MS-spectra these metabolites (Leu and Ile, TDCA and TCDCA, GCDCA, and GDCA, ADMA, and SDMA) were quantified together.

### 2.3. Method Validation

The quantitative performance of the developed UHPLC-ESI-MS/MS method was evaluated with respect to (a) limit of detection (LOD), (b) limits of quantitation (LOQ), (c) linearity (*R*^2^) and linear range, and (d) intra- and inter-day repeatability (Table 4). LOQs were determined as the lower and upper limits of quantitation (LLOQ and ULOQ), reported also as linear range, according to guidelines of International Council for Harmonisation of Technical Requirements for Pharmaceuticals for Human Use (ICH). The LODs (at *S/N* ≥ 3) were measured from standard samples and are remarkably different for different analytes, with the lowest LOD being < 2.5 ng mL^−1^ (being the lowest measured concentration) for Ala, AzelA, GCDCA, GDCA, Leu, Ile, N-MNA, Phe, and TCA. These results indicate an acceptable sensitivity, as the typical concentrations of the target compounds in human blood samples have a large range and the sensitivity of the method developed here is within the concentration ranges normally detected in blood samples [35]. Calibration curves and the intra- and inter-day repeatability were determined by using normalized peak areas. For the analytes which were quantified together (i.e., GCDCA and GDCA, ADMA and SDMA, and TDCA and TCDCA), only one ISTD was used. The ISTDs used for GCDCA and GDCA, ADMA and SDMA, and TDCA and TCDCA were GDCA-d6, ADMA-d7, and TCDCA-d9, respectively. Additionally, for three analytes (i.e., GBB, Crea, and β-OHB), the ISTD signal was not repeatable and; therefore, the validation parameters of these analytes were measured without normalization to an ISTD. The calibration curves were determined within a concentration range of 2.5–75,000 ng mL^−1^. The linear ranges showed a broad variation between the different analytes (Table 3). The coefficients of determination (*R*^2^) were within the accuracy demand of 80–120% and they were higher than 0.97 for all analytes and above 0.99 for most analytes.

For the repeatability studies, three standard samples (c = 100, 1000, and 10,000 ng mL^−1^) were analyzed in four consecutive runs and in three runs on five consecutive days for intra-day and inter-day repeatability measurements, respectively. Relative standard deviations (%RSD) were calculated for both the intra- and inter-day studies (Table 3). The %RSDs for the intra-day repeatability studies were generally below 1.5% and 20.8% for the retention times and normalized peak area ratios, respectively. There are a few exceptions to these results for the analytes with no internal standards (Crea, GBB, and β-OHB). The %RSDs for the intra- and inter-day repeatability for these three analytes was between 17.8% and 20.9% and between 3.5% and 24.5%, respectively.

### 2.4. Feasibility of the Method for the Analysis of Samples from a Diabetes Cohort

In total, 50 samples were selected from a previously-described study cohort of a total of 676 participants who has a wide range of albuminuria [36]. The subset was created with computational sampling, aiming at finding a small random subset of the cohort, where the distributions of potentially confounding clinical variables are as similar as possible between the two study groups. This allowed us to study associations between metabolites and albuminuria even in this small sample set whilst avoiding the confounding effects of other factors. The clinical variables assessed were age, antihypertensive medication, BMI, duration of diabetes, glycated hemoglobin (HbA1c), insulin day dose, sex, smoking, systolic blood pressure, total cholesterol, and total triglycerides.

Selection of the best random subsample was done in four steps: (1) In total, 1 million *N* = 25 + 25 sub-samples were drawn with random sampling, (2) the correlation between each clinical variable and the albuminuria group variable was computed for each subsample, (3) the highest absolute value of correlation in each subsample was identified, and (4) the random subsample with the lowest value of maximum correlation was selected for being the least-confounded random subset for analysis.

Computational selection resulted in a balanced subset of samples from 25 normo-albuminuric and 25 macro-albuminuric participants. The highest Pearson correlation to the albuminuria group variable among the clinical variables was 0.21 for total triglycerides. All other clinical variables had a lower absolute correlation to the group variable, suggesting that the selected small subset was not confounded by imbalance in the clinical characteristics.

Associations between metabolite concentrations and relevant clinical variables were tested with metabolite-specific mixed-effects models using the R-package limma [37]. Metabolite concentrations entered the model as the dependent variable, participant identity as the random effect and the following clinical variables as fixed effects: albuminuria group, age, BMI, estimated globular filtration rate (eGFR; kidney function), glycated hemoglobin (HbA1c; glycemic control), sex, systolic blood pressure, total cholesterol, total triglycerides. Significance tests of coefficients were corrected for multiple testing over the metabolites with the Benjamini–Hochberg method [38].

**Figure 2:**
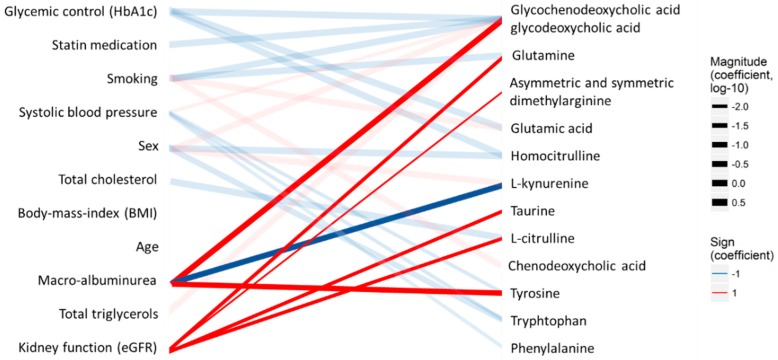
Associations between clinical measurements (left) and the quantified analytes (right) in the type 1 diabetes T1D cohort. The lines indicate statistical associations (red—positive association and blue—inverse/opposite association; line width—strength of the association). Associations directly related to diabetic kidney disease are highlighted with bold lines.

Associations indicated by significant model coefficients (multiple-testing-corrected *p* < 0.05) were visualized as a bipartite network (Figure 2) between clinical variables and metabolites with the R-package ggplot2 [39]. Strength (log-10-transformed coefficients) and the signs of each association were shown in the width and the color of the line, respectively. Metabolomic associations to albuminuria group and eGFR, which are the key variables of the present study, were highlighted with opaque lines.

The target panel included metabolites which have previously been associated particularly with kidney functions. The analysis resulted in concentrations of the measured metabolites in 50 participants with T1D. For the statistical analyses, only metabolites that were detected in over 70% of the samples were included, resulting in 20 metabolites. Macro-albuminuria, which is an indicator of kidney disease, was associated with elevated GCDCA and GDCA, Tyr, Trp, and decreased Kynu (Table 4, Figure 2). Estimated globular filtration rate (eGFR; kidney function), was associated with ADMA and SDMA, Cit, Gln, taurine, and Tyr. Glycated hemoglobin (HbA1c; glucose control) was associated with decreased GCDCA and GCDA, Glu, and HCit. Smoking was associated with elevated Glu and decreased Gln as well as to a disruption in the balance of the bile acids GCDCA and GDCA. Although no metabolomic associations were found with age or BMI in this small sub-study, several metabolites were associated with sex, statin medication, systolic blood pressure, total cholesterol, and total triglycerides. It should; however, be noted that as our target panel is based on reported markers of (pre)diabetes and diabetic complications, and does not cover the entire metabolome, a comprehensive pathway analysis could be biased and not fully reliable. The quantitative results are presented in Table 4.

## 3. Discussion

The main goal in the selection of conditions for sample preparation was the development of a workflow that is simple, robust, and feasible to automate, while taking into consideration the LC-MS method as well. The optimized sample preparation procedure, including the derivatization of amino acids and related compounds, was fast, and, by optimization of the solvent composition, we could improve the sensitivity and robustness of the derivatization step in comparison with the conventional derivatization procedures. Overall, the sample preparation is very fast, as the derivatization takes place immediately after addition of the reagent and all steps of the sample preparation can be done with automated robotic sample preparation systems. The advantage of the derivatization is that it increases the retention of the amino acids and thus allows the use of reversed-phase LC, which is more robust than, for example, hydrophilic interaction chromatography, particularly when the goal is to simultaneously analyze very polar (e.g., small amino acids) and relatively non-polar compounds (e.g., bile acids).

The linear range of the method as well as the LODs were in the range of the biological concentrations typically detected in blood-based samples. This shows that the method has both good linearity and quantitation ability for each analyte, with accuracies well within the general requirement of 80–120%. Moreover, the method developed here proved to be fast (with a sample analysis time of less than 10 min) and robust. Thus, in terms of throughput, the method is suitable for large-scale analysis. Currently, LC-MS techniques are applied in endocrinology, screening for inborn errors of metabolism, therapeutic drug monitoring/toxicology confirmation, vitamin analysis, and, more recently, the peptide and protein quantitation [40]. It should be noted that introducing a LC-MS/MS method into patient care requires that the methodology should undergo rigorous and systematic validation, including all steps of the analytical workflow, starting from the chemicals, solvent quality, columns and maintenance of the system to data processing and interpretation, in addition to traditional validation parameters that have been covered here. It should be also noted that trained personnel is a prerequisite in the use of LC-MS in clinical laboratory.

The feasibility of the developed UHPLC-ESI-MS/MS method for the analysis of biological samples was demonstrated by analyzing plasma samples from individuals with diabetes who had a wide range of albuminuria. Albuminuria is a pathological condition where the protein albumin is present in the urine in abnormal amounts. In healthy subjects (normo-albuminuric), only trace amounts of albumin (<30 mg/24 h) are present in the urine while subjects with elevated amounts of albumin in the urine, on the other hand, can be classified as either micro-albuminuric (c = 30–299 mg/24 h) or macro-albuminuric (c ≥ 300 mg/24 h) [41]. Albuminuria is a sign of diabetic kidney disease, which often occurs especially in subjects with type 1 diabetes [36,41,42]. In type 2 diabetes (T2D), microalbuminuria is an independent risk factor for the prevalence of diabetic retinopathy [42,43]. In the general population, predictors of incident albuminuria include age, male sex, smoking, and low HDL cholesterol level [44].

Several target metabolites showed either up- or downregulation in the T1D patients with albuminuria, although not all differences reached statistical significance. More specifically, we observed that several of the metabolites showed statistical associations related to the measured kidney functions and eGFR. We observed alterations in many of the amino acids measured, although not all changes reached statistical significance. Our results agree with a recent study that showed altered plasma amino acid profiles in DKD, showing that tyrosine was significantly increased in T2D patients with microalbuminuria [45]. Tyrosine has, in a recent meta-analysis, been shown to be one of the risk factors for T2D with 36% increased risk [46]. Several studies have indicated that abnormal amino acids levels are associated with diabetic kidney disease, although with somewhat contrary patterns of amino acids [28,35,45,47]. Indeed, changed amino acids metabolites might actually be more contributable to the dysregulated renal filtration state, which is unlikely to be revealed in the early pathologies of DKD, as suggested in a recent systematic review of metabolic biomarkers of DKD [28]. We also observed associations between the eGFR and ADMA, glutamine, taurine, and citrulline, in agreement of several previous studies [16,48]. Particularly, ADMA has been suggested as a candidate biomarker for diabetic kidney complications, whilst elevated levels of ADMA have been shown to predict a more accelerated course of renal function loss and promoted the development of renal damage [15,16,48]. Bile acids which have important roles as signaling molecules controlling glucose, lipid, and energy metabolism were significantly different in subjects with macro-albuminuria, and they were further associated with glycemic control. Altered bile acid metabolism has been observed particularly in T2D patients [49], but there are no earlier studies of bile acids metabolism in subjects with albuminuria. Interestingly, the main possible confounders previously linked with albuminuria, both in the general population and in diabetic patients, such as smoking, sex, or age, showed no significant associations with the metabolites most strongly linked with macro-albuminuria or kidney functions. Overall, our results suggest that the developed analytical method is feasible for performing targeted metabolomic analysis of plasma samples from diabetic patients, and that it can be used for more accurate stratification of diabetic patients—making it; thus, suitable for the use in the diabetes clinic.

Validation of the method showed that the selected panel of markers can be effectively used for classification of subjects with diabetic complications, such as macro-albuminuria. However, several of the metabolites in the current panel are related to a wide range of complications, both in T1D and T2D. Further evaluation of the clinical relevance of the method is clearly needed, in order to evaluate the full potential of this diagnostic panel in the stratification of prediabetes, metabolic, and diabetic complications.

## 4. Materials and Methods

### 4.1. Chemicals and Standard Solutions

LC-MS grade water (H_2_O), methanol (MeOH), isopropanol (IPA), and acetonitrile (ACN) were purchased from Honeywell International Inc. (Morristown, NJ, USA). HPLC grade dichloromethane (DCM), anhydrous ACN, analytical grade formic acid (HCOOH), and reagent grade potassium carbonate (K_2_CO_3_), potassium bicarbonate (KHCO_3_), sodium hydroxide (NaOH), hydrochloric acid (HCl), and 5-sulphosalisylic acid dehydrate (SSA) were purchased from Sigma-Aldrich (Steinheim, Germany). 6-aminoquinoline-N-hydroxy-succinimidyl carbamate (AQC) for derivatization of amino acids was purchased from Santa Cruz Biotechnology, Inc. (Dallas, TX, USA).

Stock solutions (4.0 mg mL^−1^) of the analytes and internal standards (Table 1 and Table 5) were prepared by dissolving in 0.1 M HCl, H_2_O, H_2_O:MeOH (90:10, *v*/*v*) or in MeOH and further diluting them with 0.6 M carbonate buffer (pH 8.9) and 1 M NaOH (3:1, *v*/*v*) (in order to subsequently neutralize and adjust the pH) to the following concentration levels: 2.5, 5.0, 7.5, 10.0, 25, 50, 75, 100, 250, 500, 750, 1000, 2500, 5000, 7500, 10,000, 25,000, 50,000, and 75,000 ng mL^−1^. A total of 20 µL of an internal standard solution (ISTD MIX) containing each of the internal standards (Table 5) was added to all samples. The samples were vortex mixed and 20 µL of a 5 mg mL^−1^ AQC-reagent, which was dissolved in anhydrous ACN (at 55 °C) was added for derivatization of the amino acids and related metabolites (Supplementary Figure S4). Finally, the samples were vortex mixed and stored at −80 °C until analysis. The calibration curves were constructed using at least five measuring points and linear regression with 1/x weighing. For α(R)-OHB and α(S)-OHB, only three measuring points could be used due to the high LOD of these analytes.

### 4.2. Samples

Plasma samples from a previously-described cohort [30,36] were used for validation of the method. In short, during 2009–2011, a total of 1285 patients were invited to enter a study examining diabetic complications at the Steno Diabetes Center Copenhagen (SDCC). The study conformed to the Declaration of Helsinki and was approved by the Danish National Committee on Biomedical Research Ethics (2009-056; NCT01171248). Additionally, all patients gave written, informed consent. Of the invited 1285 patients, 676 accepted to participate and for our purposes, to demonstrate method functionality, a subset of 50 patient samples was analyzed. In addition to these plasma samples, pooled plasma samples from the SDCC were used for method development and validation as well as for quality control. All plasma samples were stored at −80 °C until analysis.

### 4.3. Sample Preparation

Sample preparation included protein precipitation and derivatization (see 4.1. for details of standards, stock solutions and derivatization reagent). A total of 10 µL of 1 M 5-sulphosalisylic acid dehydrate (SSA) solution was added to 30 µL of plasma sample, samples were vortex mixed and centrifuged at 9000 RCF (5 min at 4 °C) after which 20 µL of the upper phase was collected. After, addition of 20 µL of the ISTD MIX 20 µL of a 6-aminoquinoline-N-hydroxy-succinimidyl carbamate-reagent (AQC-reagent) (5 mg mL^−1^, at 55 °C) was added, and the samples were vortex mixed and stored at −80 °C until analysis.

The samples in the validation study were randomized before sample preparation and again before analysis. Calibration curves were created at the beginning and at the end of the sample analyses. Additionally, blank samples and pooled plasma samples were included in the analytical sequence for quality control purposes. Samples were injected three times, resulting in three technical replicate measurements for each of the 50 samples.

### 4.4. Ultra High-Performance Liquid Chromatography (UHPLC)-Mass Spectrometry

The UHPLC system was 1290 Infinity system from Agilent Technologies (Santa Clara, CA, USA) and it was equipped with a multi-sampler (maintained at 10 °C), a binary solvent manager, and a column thermostat (maintained at 40 °C). The multi-sampler was set to utilize the multi-wash option as the needle wash. Here two mixtures, ACN:MeOH:IPA:H_2_O (1:1:1:1, *v*/*v*/*v*/*v*) + 0.1% HCOOH and 10% DCM in MeOH, were used for 8 s after each injection in order to clean the needle and the needle seat. Finally, the needle and the needle seat were flushed with the initial gradient conditions for 8 s. Separations were performed on a Kinetex^®^ F5 column (100 × 2.1 mm, particle size 1.7 µm) from Phenomenex (Torrance, CA, USA) with a flow rate of 0.4 mL min^−1^ and an injection volume of 2 µL. H_2_O + 0.1% HCOOH (A) and ACN:IPA (2:1, *v*/*v*) + 0.1% HCOOH (B) were used as the mobile phases for gradient elution. The gradient was as follows: from 0 to 1 min 1% B, from 1 to 1.8 min 1–18% B, from 1.8 to 3.4 min 18–21% B, from 3.4 to 7 min 21–65% B, from 7 to 7.1 min 65–100% B and from 7.1 to 8.9 min 100% B. Each run was followed by a 2.5 min re-equilibration period under initial conditions (1% B).

The mass spectrometer was a 6460 triple quadrupole system from Agilent Technologies. It was interfaced with an Agilent Jet Stream electrospray ionization source. The analytes were ionized in positive or in negative ion mode depending on the properties of the analyte. Nitrogen generated by a Genius 3010 nitrogen generator from PEAK Scientific Instruments Ltd. (Inchinnan, Scotland, UK) was used as the nebulizing gas (pressure 29 psi) and as the sheath gas at 250 °C and 6 L min^−1^ and at 310 °C and 9 L min^−1^, respectively. Pure nitrogen (6.0) from Praxair (Fredericia, Denmark) was used as the collision gas. The capillary voltage was set to 3000 V and the nozzle voltage to 1000 V. MS- and MS/MS-spectra (scan range *m/z* 40–600) were acquired for each analyte to select the best precursor and product ions for selected reaction monitoring (SRM) analyses. The fragmentor voltages, collision energies (CE), and cell accelerator voltages were separately optimized for each ion transition of the analytes (Table 3) and the internal standards (Table 4). MassHunter LC/MS Data Acquisition Software (version B.08.02) was used for all data acquisition. For data processing different software were used: MassHunters Quantitative Analysis Software (version B.07.00), Skyline Daily (version 4.1) [50], and R [51].

Data from the diabetes cohort were processed as follows: (i) Peaks were picked in Skyline [50], (ii) resulting peak areas were normalized to matching internal standard peak areas in R, and (iii) the resulting peak area ratios were calibrated to concentrations in R based on metabolite-specific calibration curves run during the analysis sequence.

## Figures and Tables

**Table 1:** Standard compounds acquired for quality control and for quantitation.

Compound	Abbreviation	Group	Vendor	Solvent, Stock Solution
L-Glutamine	Gln	Amino acids + related metabolites	Sigma-Aldrich	H_2_O
Glycine	Gly			0.1 M HCl
L-Alanine	Ala			
L-Leucine	Leu			
L-Isoleucine	Ile			
L-Phenylalanine	Phe			
L-Tryptophan	Trp			
L-Tyrosine	Tyr			
L-Glutamic Acid	Glu			
L-Citrulline	Cit			
L-Homocitrulline	HCit		SCB	
Asymmetric dimethylarginine	ADMA			
Symmetric dimethylarginine	SDMA			
DL-2-Aminoadipic Acid	AADA		Sigma-Aldrich	
L-Kynurenine	Kynu			
Taurine	Taurine			
Deoxycholic Acid	DCA	Bile acids	Sigma-Aldrich	MeOH
Glycochenodeoxycholic Acid	GCDCA			
Glycodeoxycholic Acid	GDCA			
Glycocholic Acid	GCA			
Taurodeoxycholic Acid	TDCA			
Taurochenodeoxycholic Acid	TCDCA			
Deoxychenocholic Acid	CDCA			
Cholic Acid	CA			
Taurocholic Acid	TCA			
Glycoursodeoxycholic Acid	GUDCA		CIL	
Ursodeoxycholic Acid	UDCA			
Tauroursodeoxycholic Acid	TUDCA			
Creatinine	Crea	Other metabolites	Sigma-Aldrich	10% MeOH
Indoxyl Sulfate	IndS			
N-methyl-nicotinamide	N-MNA		SCB	
Gamma-butyrobetaine	GBB			
Azelaic Acid	AzelA	Small organic acids	Sigma-Aldrich	MeOH
L-3-hydroxybutyric Acid	β-OHB			10% MeOH
R-2-hydroxybutyric Acid	α(R)-OHB			
S-2-hydroxybutyric Acid	α(S)-OHB			

Vendor information: Sigma-Aldrich (Steinheim, Germany); SCB: Santa Cruz Biotechnology, Inc. (Dallas, TX, USA); CIL: Cambridge Isotope Laboratories Inc. (Tewksbury, MA, USA).

**Table 2:** Optimized fragmentor voltages, collision energies (CE), and cell accelerator voltages for each ion transition of the analytes and internal standards. The ion transition used for quantification is marked with an *.

Compound	Molecular Weight (MW)	Ion Transition	Polarity	Fragmentor Voltage (V)	Collision Energy (V)	Cell Accelerator Voltage (V)
AADA	161.2	330.2–160.1	Negative	150	10	1
ADMA and SDMA	202.3	371.2–201.2 *	Negative	150	5	5
		371.2–156.1	Negative	150	20	1
Ala	89.1	258.1–88.1	Negative	100	15	3
AzelA	188.2	187.2–169	Negative	150	10	1
		187.2–125.2 *	Negative	150	15	1
β-OHB	104.1	103.2–59.2	Negative	100	5	1
CA	408.6	407.3–407.3 *	Negative	250	0	1
		407.3–343.3	Negative	250	35	3
CDCA	392.6	391.3–391.3	Negative	250	0	3
Cit	175.2	344.4–174.2	Negative	150	4	7
Crea	113.1	114.1–86.2	Positive	150	11	4
		114.1–44.1 *	Positive	150	15	4
DCA	392.6	391.2–345.3 *	Negative	200	35	4
		391.2–327.2	Negative	200	40	4
GBB	146.2	147.2–88.1 *	Positive	100	16	1
		147.2–60.2	Positive	100	13	1
GCA	465.6	464.3–402.1	Negative	250	40	4
		464.3–74.1 *	Negative	250	45	7
GCDCA	449.6	448.3–386.3	Negative	150	40	2
GDCA	449.6	448.3–402.1	Negative	250	40	2
GCDCA and GDCA	449.6	448.3–74.2	Negative	200	55	2
Gln	146.1	315.3–145.1	Negative	100	9	6
Glu	147.1	316.1–146.1	Negative	100	6	6
Gly	75.1	244.1–74.1	Negative	200	7	4
GUDCA	449.6	448.3–386	Negative	250	40	2
		448.3–74.1 *	Negative	250	45	2
HCit	189.2	358.3–188.1	Negative	200	10	1
		358.3–145 *	Negative	150	25	2
IndS	213.2	212–132 *	Negative	100	15	2
		212–80	Negative	100	20	2
Kynu	208.2	377–316.1	Negative	150	5	2
		377–207 *	Negative	150	5	5
Leu and Ile	131.2	300.2–130.2	Negative	100	10	1
N-MNA	136.2	137.1–108.1	Positive	100	15	2
		137.1–80.2 *	Positive	100	26	2
Phe	165.2	334.2–164	Negative	100	10	1
Taurine	125.2	294.1–124.1 *	Negative	100	10	2
		294.1–80.1	Negative	100	55	2
TCA	515.7	514.3–123.8	Negative	300	65	5
		514.3–80.2 *	Negative	300	95	1
TDCA and TCDCA	499.3	498.3–107.1	Negative	250	80	1
		498.3–80.1 *	Negative	300	90	1
Trp	204.2	373.2–203.1	Negative	150	7	2
TUDCA	499.7	498.3–107.1	Negative	300	65	5
		498.3–80.1 *	Negative	300	85	1
Tyr	181.2	350.2–180.1	Negative	100	7	5
AADA-d3	164.2	333.2–145.2	Negative	100	20	2
ADMA-d7	209.8	378–208.3	Negative	100	10	5
Ala-d4	93.1	262.1–92.1	Negative	100	5	6
α-OHB-d3	107.1	106.1–59.1	Negative	100	10	1
AzelA-d14	202.3	201.2–137.2	Negative	150	10	2
β-OHB-d4	108.1	107.1–59.1	Negative	100	5	1
CA-d4	412.3	411.3–411.3	Negative	250	0	3
CDCA-d4 and DCA-d4	396.6	395.2–395.2	Negative	300	0	4
Cit-d4	179.2	348.1–135.1	Negative	100	25	2
Crea-d5	118.2	119.2–49.3	Positive	100	20	1
GBB-d9	154.7	155.2–87.3	Positive	100	15	6
GCA-d4	469.6	468.3–74.1	Negative	250	45	1
GCDCA-d4 and GUDCA-d4	453.6	452.3–74.1	Negative	250	40	1
GDCA-d6	455.7	454.3–408.2	Negative	250	55	4
Gln-d5	151.2	320.1–150.1	Negative	100	5	1
Glu-d5	152.1	321.1–151.1	Negative	100	5	1
Gly-13C,d2	78.1	247–77.1	Negative	100	5	7
HCit-2H4	193.2	362.2–192.2	Negative	100	5	6
IndS-d4	217.3	216–136.1	Negative	100	15	2
Kynu-13C6	214.2	383.1–195.8	Negative	100	10	6
Leu-d10 and Ile-d10	141.2	310.1–140	Negative	125	10	2
N-MNA-d4	140.2	141.2–84.2	Positive	100	20	7
Phe-d5	170.2	339.1–169.1	Negative	150	5	1
Taurine-d4	129.2	298.3–128.2	Negative	100	10	3
TCA-d4	519.7	518.3–80	Negative	340	100	7
TCDCA-d9	508.3	507.4–80.1	Negative	300	95	1
Trp-d8	212.3	381.2–211.2	Negative	100	10	5
TUDCA-d4	503.7	502.3–80.1	Negative	300	100	1
Tyr-d7	188.2	357.1–187.2	Negative	100	10	1
UDCA-d4	396.6	395.3–395.3	Negative	250	0	4

**Table 3:** Linearity (*R*^2^) with lower and upper limits of detection (LLOD and ULLOQ), linear range, repeatability of retention times (Rt) and intra- and inter-day repeatability of concentrations at different concentrations.

Compound	Linearity (*R*^2^) Range (LLOQ-ULOQ) (ng mL^−1^)	LOD (ng/mL^−1^)	%RSD_Rt, Intra-Day	%RSD_Area, Intra-Day (*N* = 4)			%RSD_Rt, Inter-Day	%RSD_Area, Inter-Day (*N* = 15)		
				100 ng mL^−1^	1000 ng mL^−1^	10,000 ng mL^−1^		100 ng mL^−1^	1000 ng mL^−1^	10,000 ng mL^−1^
AADA	0.984 5000–75,000	500	0.2 (*N* = 4)	-	-	9.1	0.1 (*N* = 15)	-	-	8.7
ADMA and SDMA	0.992 2500–50,000	500	0.2 (*N* = 8)	-	5.6	0.8	0.2 (*N* = 30)	-	8.5	4.2
Ala	0.996 500–50,000	<2.5	0.2 (*N* = 8)	-	4.5	3.0	0.1 (*N* = 30)	-	9.8	13.6
AzelA	0.995 500–10,000	<2.5	0.5 (*N* = 8)	-	11.4	3.9	-	-	15.8	8.4
β-OHB	0.970 2500–75,000	75	0.6 (*N* = 4)	-	-	20.9	1.2 (*N* = 15)	-	-	24.5
CA	0.996 10–10,000	7.5	0.2 (*N* = 12)	2.4	3.1	5.2	0.7 (*N* = 45)	20.8	18.1	20.2
CDCA	0.999 25–2500	7.5	0.2 (*N* = 8)	4.0	4.9	-	0.2 (*N* = 45)	4.3	5.1	14.2
Cit	0.984 500–10,000	250	0.2 (*N* = 8)	-	7.7	6.7	0.2 (*N* = 30)	-	9.1	8.3
Crea	0.973 250–7500	25	0.8 (*N* = 4)	-	17.8	-	0.0 (*N* = 15)	-	3.5	-
DCA	0.996 5–2500	2.5	0.2 (*N* = 8)	5.8	6.1	-	0.3 (*N* = 30)	4.3	8.3	-
GBB	0.974 250–10,000	50	0.5 (*N* = 8)	-	18.7	15.9	1.5 (*N* = 30)	-	27.3	28.5
GCA	0.997 50–25,000	25	0.1 (*N* = 12)	4.6	4.2	4.2	0.4 (*N* = 45)	6.8	5.1	7.3
GCDCA and GDCA	0.997 25–2500	<2.5	0.2 (*N* = 8)	1.9	4.2	-	0.5 (*N* = 30)	16.4	16.1	-
Gln	0.987 750–50,000	5	0.1 (*N* = 8)	-	5.0	7.7	0.5 (*N* = 30)	-	10.5	11.5
Glu	0.990 750–75,000	500	0.2 (*N* = 8)	-	13.9	10.7	0.3 (*N* = 30)	-	10.9	5.2
Gly	0.993 7500–75,000	1000	0.03 (*N* = 4)	-	-	16.2	0.6 (*N* = 15)	-	-	19.6
GUDCA	0.994 75–10,000	25	0.1 (*N* = 12)	5.0	9.0	10.6	0.3 (*N* = 45)	13.1	10.9	6.2
HCit	0.995 500–25,000	250	0.2 (*N* = 8)	-	8.3	2.6	0.5 (*N* = 30)	-	11.1	16.4
IndS	0.986 5000–75,000	750	0.3 (*N* = 4)	-	-	11.3	0.3 (*N* = 15)	-	-	15.4
Kynu	0.993 500–75,000	250	0.2 (*N* = 8)	-	11.2	7.4	0.4 (*N* = 30)	-	7.7	4.4
Leu and Ile	0.997 25–75,000	<2.5	0.4 (*N* = 12)	4.6	4.3	1.5	0.5 (*N* = 45)	13.0	14.0	5.7
N-MNA	0.998 25–10,000	<2.5	0.5 (*N* = 12)	1.6	6.4	3.7	1.0 (*N* = 45)	20.1	18.5	6.5
Phe	0.995 250–25,000	<2.5	0.4 (*N* = 0.4)	-	5.9	6.6	0.4 (*N* = 30)	-	10.1	4.6
Taurine	0.994 250–25,000	10	0.2 (*N* = 8)	-	8.3	5.7	0.5 (*N* = 30)	-	8.4	8.7
TCA	0.983 2500–25,000	<2.5	0.1 (*N* = 4)	-	-	4.5	0.3 (*N* = 15)	-	-	15.5
TDCA and TCDCA	0.984 1000–25,000	10	0.7 (*N* = 8)	-	0.4	5.7	0.7 (*N* = 30)	-	2.6	4.2
Trp	0.996 25–25,000	25	0.4 (*N* = 12)	9.0	2.9	4.7	0.5 (*N* = 45)	18.8	5.4	5.3
TUDCA	0.990 250–10,000	10	0.1 (*N* = 8)	-	5.5	4.5	0.7 (*N* = 30)	-	1.8	3.0
Tyr	0.992 50–75,000	25	0.2 (*N* = 12)	10.3	9.1	4.4	0.3 (*N* = 45)	5.7	8.4	3.4
UDCA	0.991 50–50,000	25	0.2 (*N* = 12)	1.5	3.5	3.3	0.2 (*N* = 45)	3.5	10.3	5.6

**Table 4:** Concentrations of metabolites in the validation cohort and their *p* values.

Metabolite Name	Normo-Albuminuria, Mean c (Standard Deviation)	Macro-Albuminuria, Mean c (Standard Deviation)	*p* Value	adj. *p* Value
Glycochenodeoxycholic Acid and Glycodeoxycholic Acid	4.33 (11.74)	2.10 (6.58)	0.00012	0.0021
L-Kynurenine	383.23 (249.28)	309.03 (86.53)	0.00043	0.0034
Tyrosine	6185.75 (1865.87)	7012.51 (2076.69)	0.00057	0.0034
Tryptophan	5913.04 (1705.38)	6388.34 (1346.28)	0.031	0.14
Asymmetric dimethylarginine and Symmetric Dimethylarginine	165.73 (51.07)	153.35 (18.60)	0.26	0.57
Leucine and Isoleucine	6393.48 (3159.65)	7303.02 (3656.17)	0.28	0.57
Chenodeoxycholic Acid	1101.07 (7.10)	1099.58 (6.38)	0.29	0.57
Glycine	9696.30 (5174.24)	10,313.80 (3604.96)	0.32	0.58
Glutamine	31,651.43 (8920.90)	29,020.85 (6798.27)	0.4	0.63
L-Citrulline	2235.88 (1160.64)	2253.08 (852.27)	0.42	0.63
Alanine	16,925.72 (4875.55)	16,087.19 (3345.81)	0.58	0.75
Indoxyl Sulfate	907.87 (493.53)	920.80 (561.30)	0.6	0.75
Homocitrulline	11.36 (25.76)	10.21 (20.90)	0.62	0.75
Taurine	4741.00 (2046.23)	4128.35 (1424.84)	0.77	0.86
Phenylalanine	9337.50 (2600.13)	8949.64 (2062.99)	0.86	0.91
Glutamic Acid	8164.60 (3588.71)	9304.01 (7562.67)	0.93	0.93

**Table 5:** Internal standards, with concentrations in ISTD MIX, acquired for quality control and for quantitation.

Internal Standard	Abbreviation	Group	Vendor	Solvent, Stock Solution	Concentration in ISTD MIX (ng mL^−1^)
d5-Glutamine	d5-Gln	Amino acids + related metabolites	CIL	H_2_O	30,000
d10-L-Leucine	d10-Leu		CDN	0.1 M HCl	5000
^2^H_4_-L-Homocitrulline	2H4-HCit		Alsachim		
Glycine-1-^13^C,2,2-d_2_	13C, d2-Gly		Sigma-Aldrich		
d4-DL-Alanine	d4-Ala				
d5-L-Glutamic Acid	d5-Glu				
d10-Isoleucine	d10-Ile		CIL		
d5-L-Phenylalanine	d5-Phe				500
d8-Tryptophan	d8-Trp				5000
d7-Tyrosine	d7-Tyr				
d4-Citrulline	d4-Cit				500
d3-L-2-Aminoadipic Acid	d3-AADA				10,000
d7-Asymmetric dimethylarginine	d7-ADMA				5000
^13^C_6_-Kynurenine	13C6-Kynu		Alsachim		30,000
d4-Taurine	d4-Taurine				500
d4-Deoxycholic Acid	d4-DCA	Bile acids	CDN	MeOH	500
d4-Glycocholic Acid	d4-GCA				250
d4-Deoxychenocholic Acid	d4-CDCA				500
d4-Glycoursodeoxycholic Acid	d4-GUDCA				5000
d4-Cholic Acid	d4-CA				500
d4-Ursodeoxycholic Acid	d4-UDCA				250
d4-Glychochenodeoxycholic Acid	d4-GCDCA		CIL		5000
d6-Glycodeoxycholic Acid	d6-GDCA				30,000
d9-Taurochenodeoxycholic Acid	d9-TCDCA				500
d4-Taurocholic Acid	d4-TCA				
d4-Tauroursodeoxycholic Acid	d4-TUDCA				250
d5-Creatinine	d5-Crea	Polar metabolites	CDN	10% MeOH	10,000
d4-N-methyl-nicotinamide	d4-N-MNA				250
d9-Gamma-butyrobetaine	d9-GBB				500
d4-Indoxyl Sulfate	d4-IndS		Sigma-Aldrich		5000
d14-Azelaic Acid	d14-AzelA	Small organic acids	CDN	MeOH	5000
d4-3-Hydroxybutyric Acid	d4-β-OHB			10% MeOH	100,000
d3-2-Hydroxybutyric Acid	d3-α-OHB				

Vendor information: Sigma-Aldrich (Steinheim, Germany); CDN: C/D/N Isotopes, Inc. (Quebec, Canada); CIL: Cambridge Isotope Laboratories Inc. (Tewksbury, MA, USA); Alsachim (Illkirch Graffenstaden, France); SCB: Santa Cruz Biotechnology, Inc. (Dallas, TX, USA).

## Supplementary Materials

The following are available online at https://www.mdpi.com/2218-1989/9/9/184/s1, Figure S1: Derivatization reaction. Figure S2: Structures of compounds of interest, amino acids, and amino acid-related compounds, Figure S3: Structures of compounds of interest, bile acids, Figure S4: Structures of compounds of interest, small organic acids and other metabolites of interest, Figure S5: (a) MS-spectrum and (b) MS/MS-spectrum of taurine, Figure S6: (a) MS-spectrum and (b) MS/MS-spectrum of azelaic acid, Figure S7: (a) MS-spectrum and (b) MS/MS-spectrum of gamma-butyrobetaine, Figure S8: (a) MS-spectrum and (b) MS/MS-spectrum of glycolic acid, Figure S9: (a) MS-spectrum and (b) MS/MS-spectrum of L-homocitrulline.
