# Supplementary material for: Targeted Clinical Metabolite Profiling Platform for the Stratification of Diabetic Patients
Source: Metabolites. 2019 Sep 14;9(9):184. doi: 10.3390/metabo9090184 (PMC6780060; doi:10.3390/metabo9090184)
Supplement: Supplementary file 1 [file metabolites-09-00184-s001.pdf]

Targeted Clinical Metabolomics Platform for the Stratification of Diabetic Patients

Linda Ahonen et al.

Supplemental Material

Supplemental Methods

Figure S1A. Structures of compounds of interest, amino acids and amino acid related compounds.

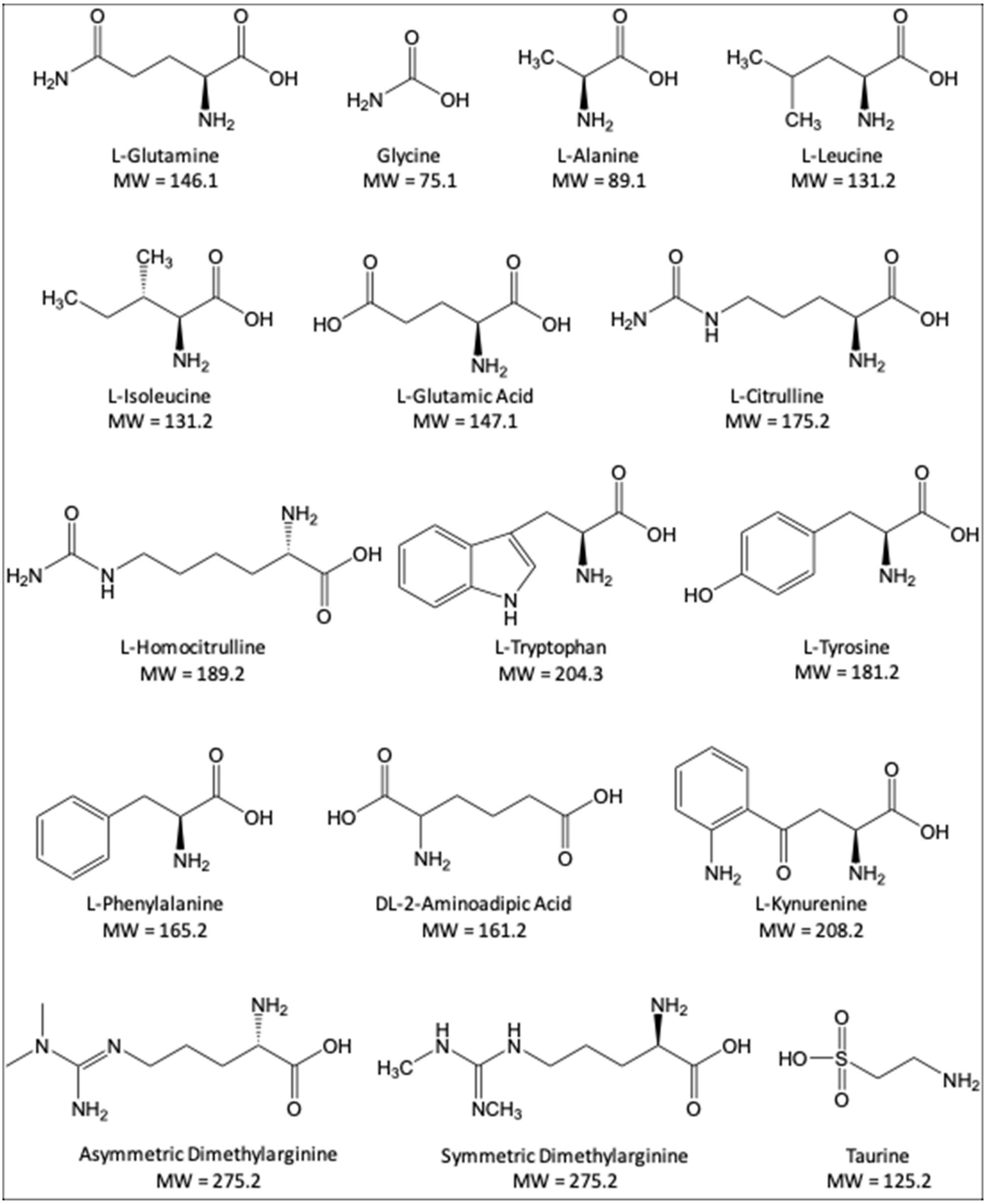

Figure S2. Structures of compounds of interest, bile acids.

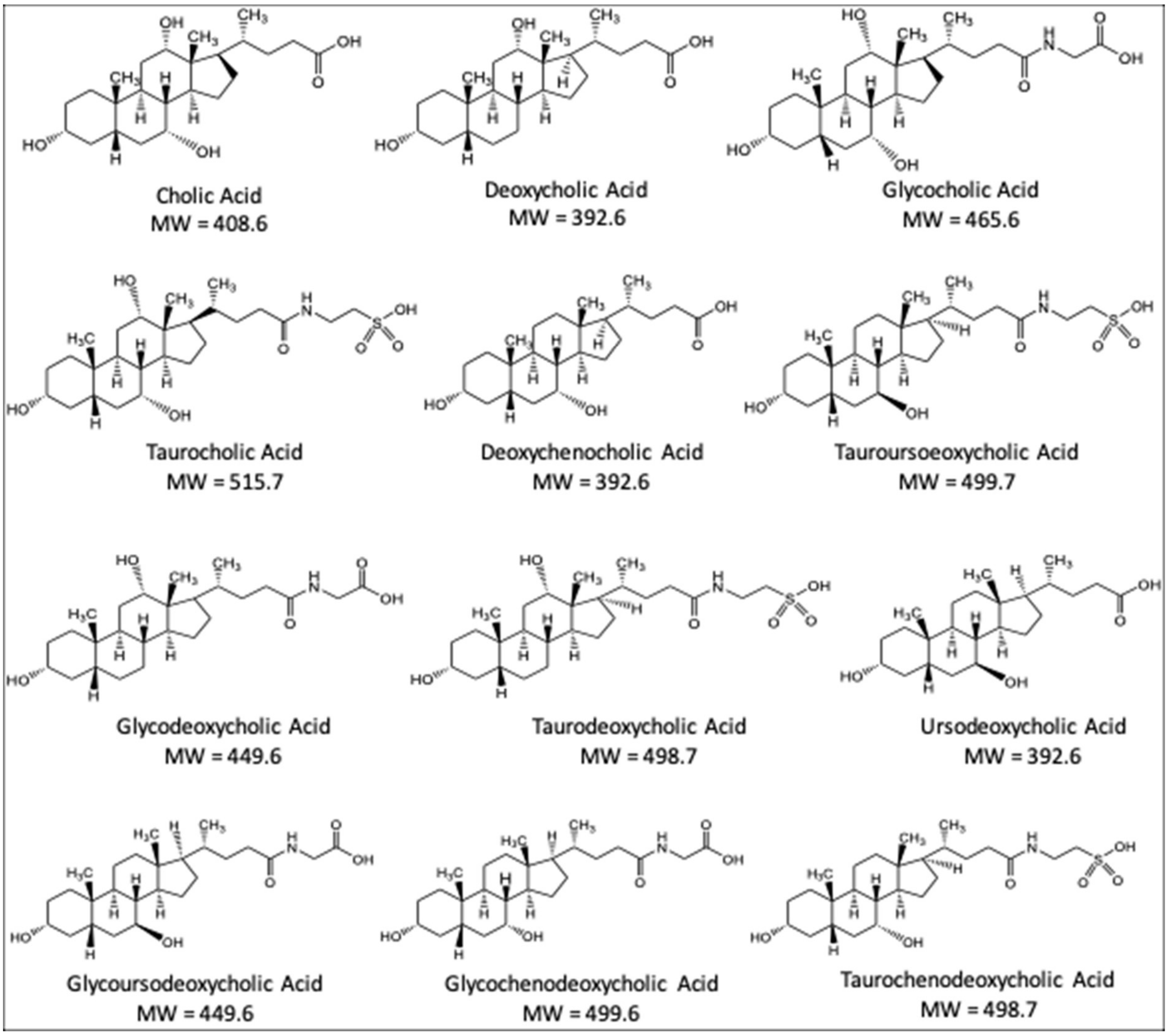

Figure S3. Structures of compounds of interest, small organic acids and other metabolites of interest.

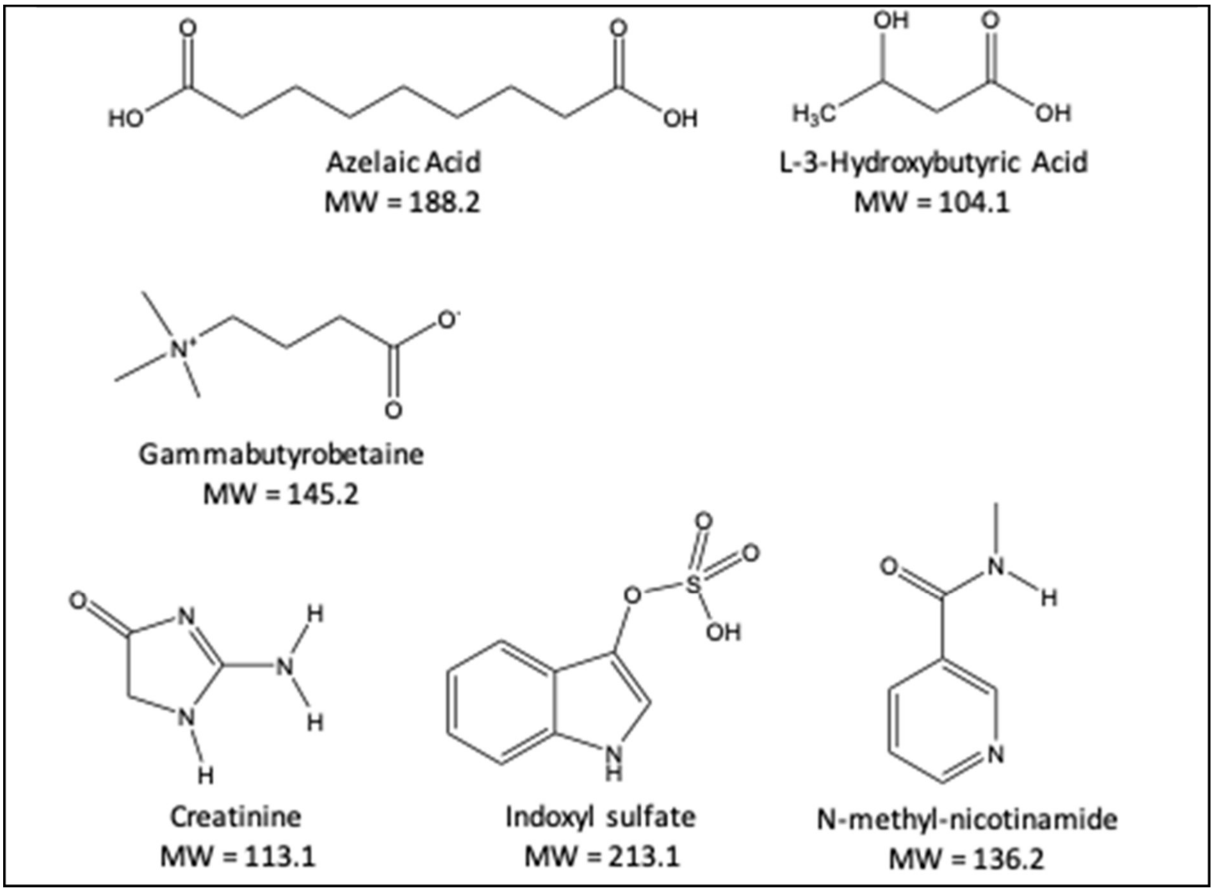

Figure S4. Derivatization reaction.

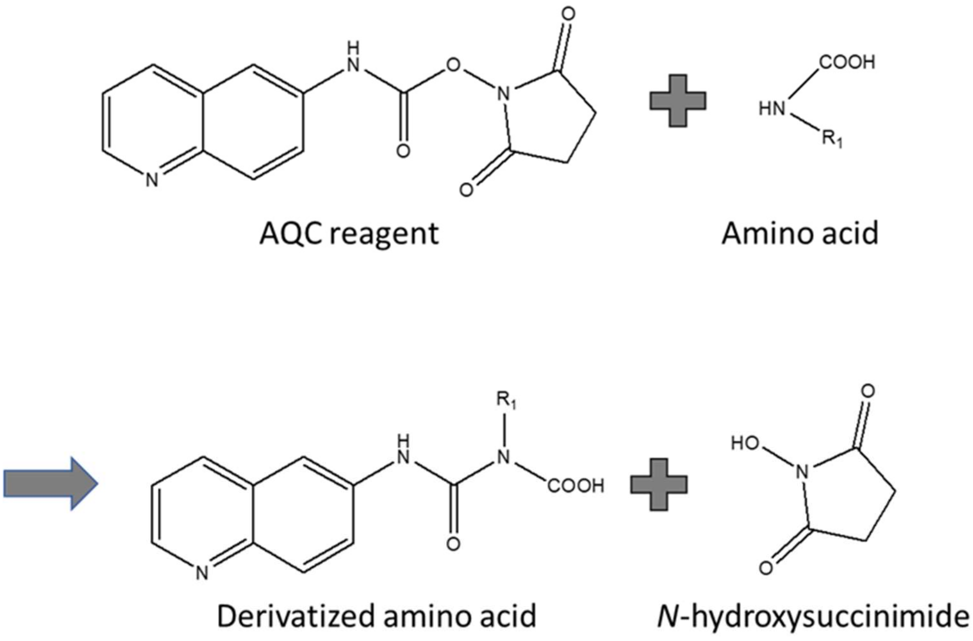
H
N
O
O
O
N
O
COOH
HN
R1
AQC reagent
Amino acid
R1
H
N
N
COOH
O
N
HO
O
N
O
Derivatized amino acid
N-hydroxysuccinimide

Figure S5. a) MS-spectrum and **b)** MS/MS-spectrum of Taurine.

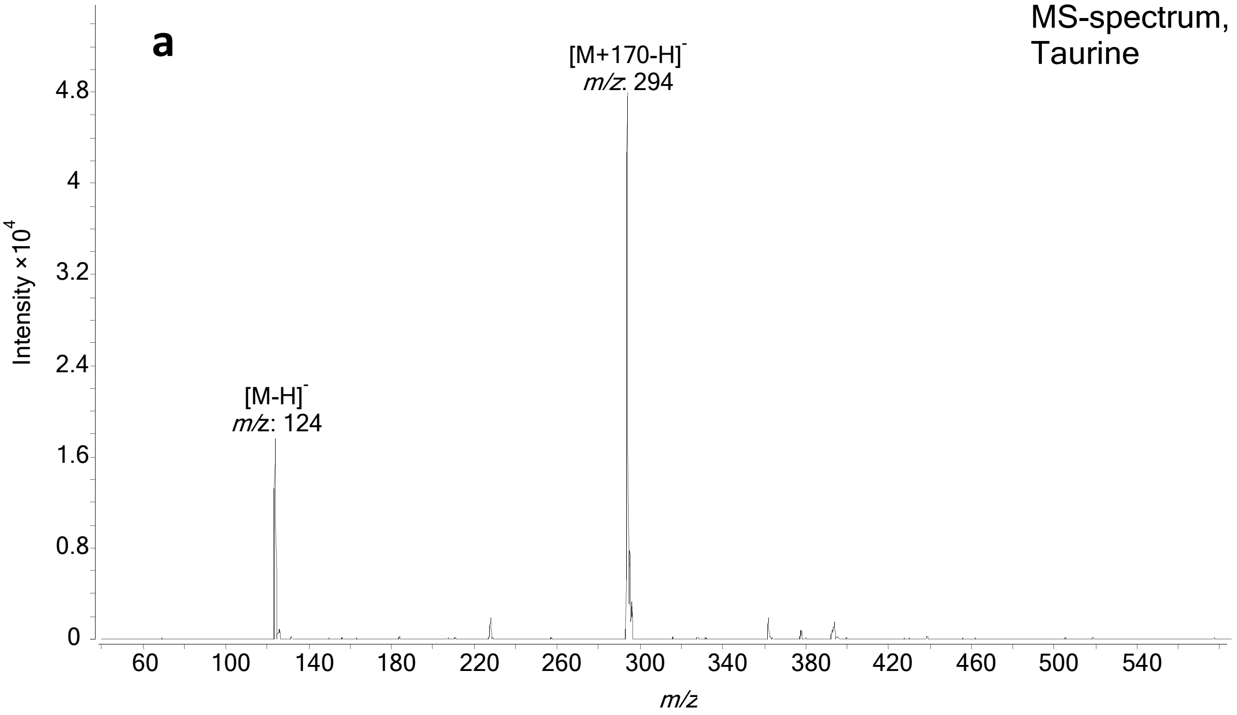

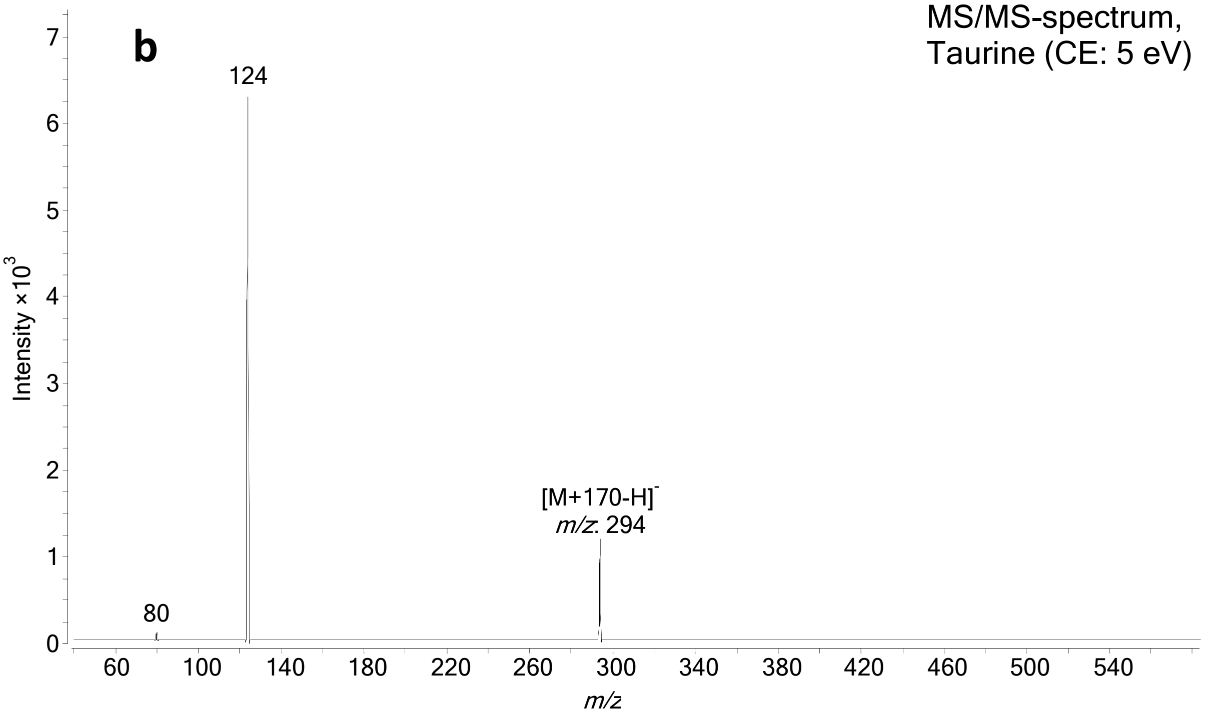

Figure S6. a) MS-spectrum and **b)** MS/MS-spectrum of Azelaic Acid.

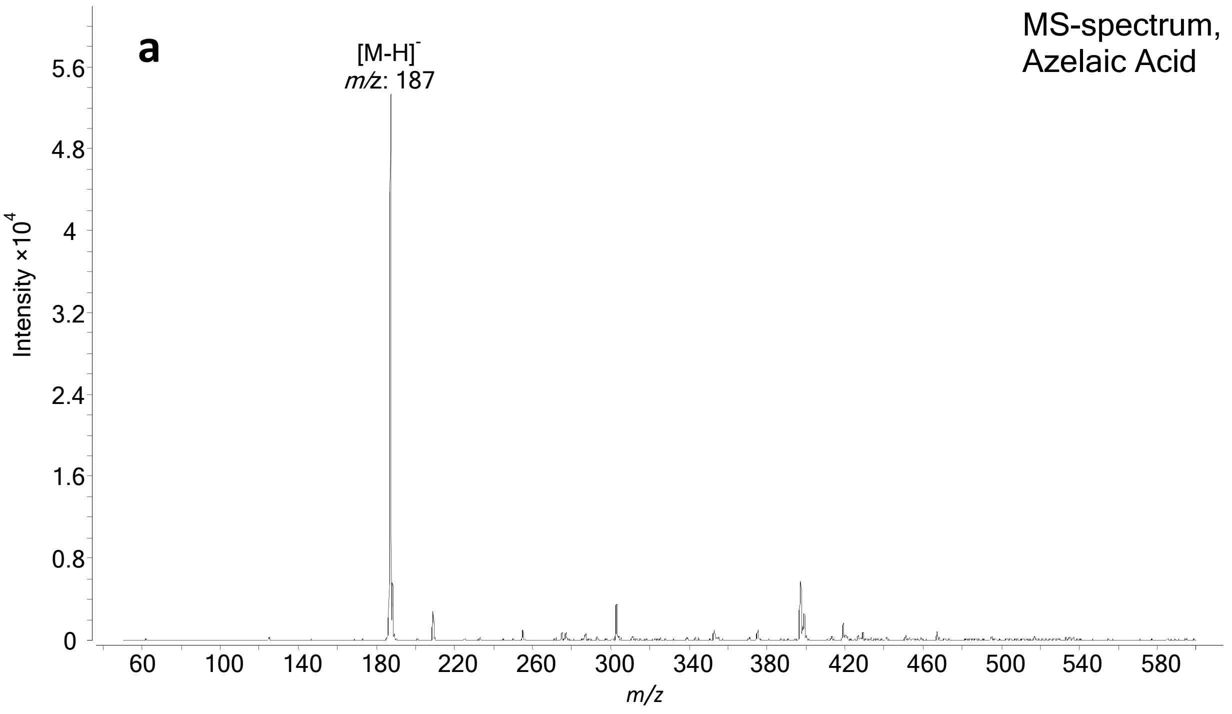

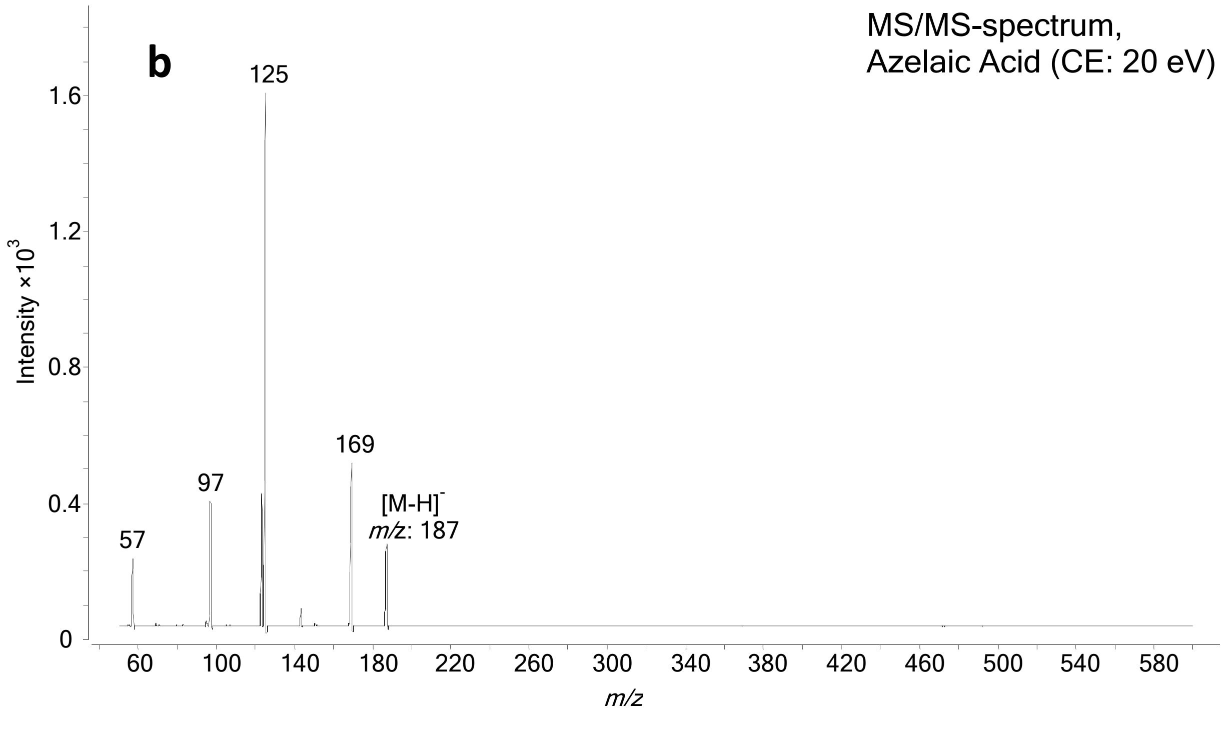

Figure S7. a) MS-spectrum and **b)** MS/MS-spectrum of Gamma-butyrobetaine.

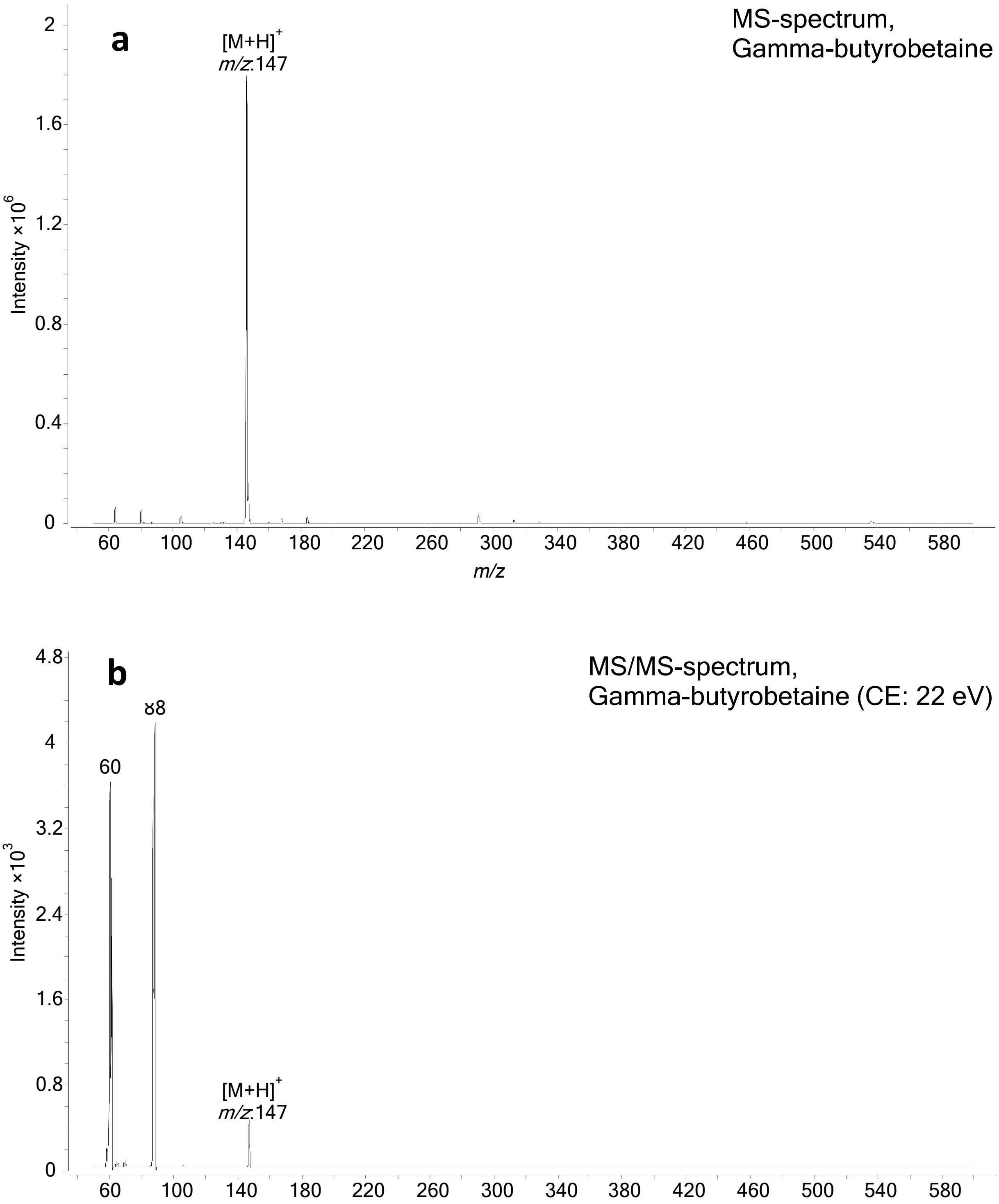

Figure S8. a) MS-spectrum and **b)** MS/MS-spectrum of Glycolic Acid.

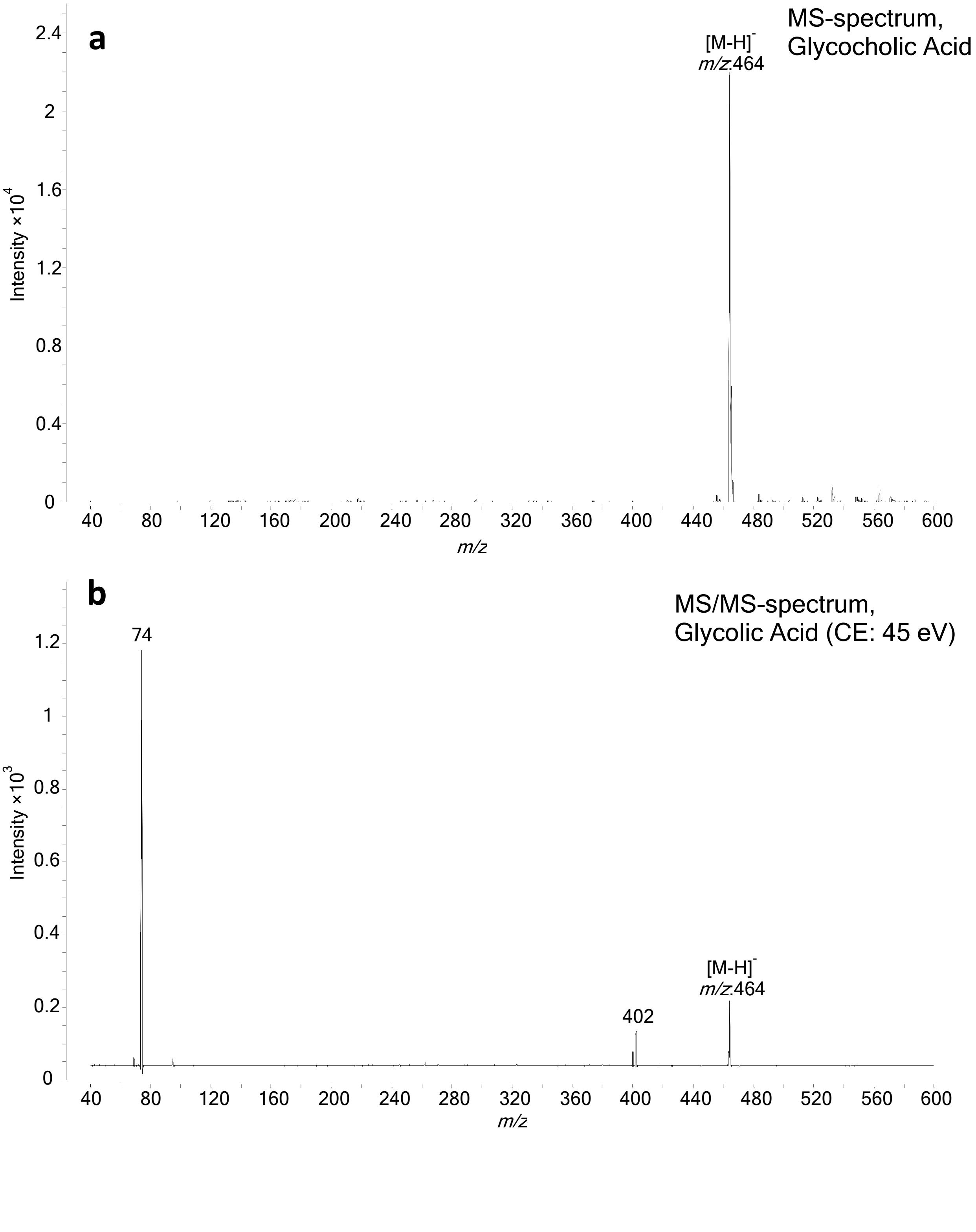

Figure S9. a) MS-spectrum and **b)** MS/MS-spectrum of L-Homocitrulline.

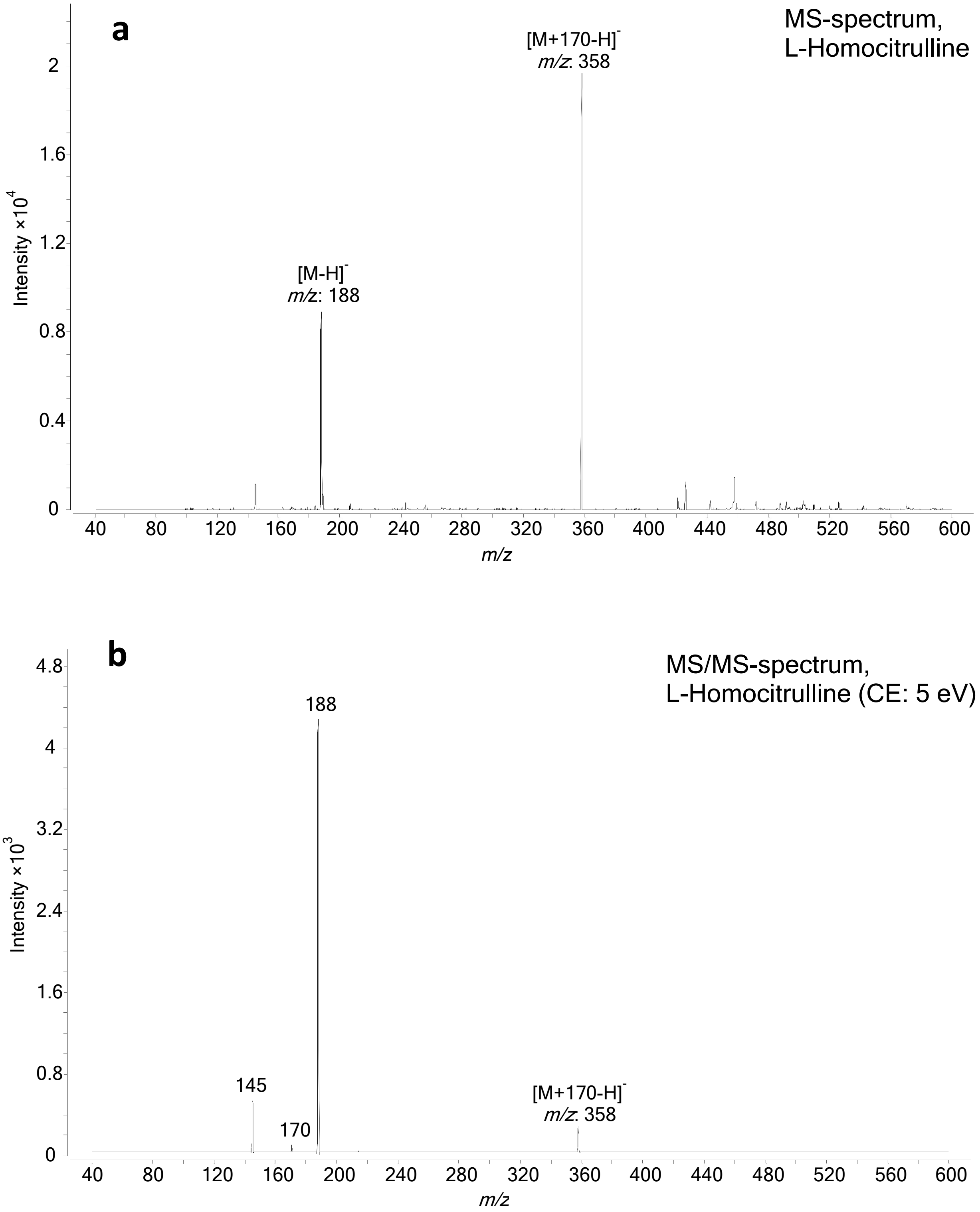

References

1. MacLean B, Tomazela DM, Shulman N, Chambers M, Finney GL, Frewen B, et al. Skyline: An open source document editor for creating and analyzing targeted proteomics experiments. Bioinformatics (Oxford, England) 2010;26:966-8.
2. Team RC. R: A language and environment for statistical computing, r foundation for statistical computing. Vienna, Austria 2017.
